# PIK3CA copy number aberration and activation of the PI3K-AKT-mTOR pathway in varied disease states of penile cancer

**DOI:** 10.1371/journal.pone.0198905

**Published:** 2018-06-14

**Authors:** Anthony Adimonye, Elzbieta Stankiewicz, Susannah La-Touche, Sakunthala Kudahetti, Giorgia Trevisan, Brendan Tinwell, Cathy Corbishley, Yong-Jie Lu, Nick Watkin, Daniel Berney

**Affiliations:** 1 Barts Cancer Institute, Centre for Molecular Oncology, Barts and The London School of Medicine and Dentistry, Queen Mary University of London, London, United Kingdom; 2 Department of Histopathology, Royal London Hospital, Barts Health NHS Trust, London, United Kingdom; 3 Department of Cellular Pathology, St George’s Hospital, London, United Kingdom; 4 Department of Urology, St George’s Hospital, London, United Kingdom; University of South Alabama Mitchell Cancer Institute, UNITED STATES

## Abstract

**Background:**

Therapeutic targeting of the PI3K-AKT-mTOR pathway may benefit patients with advanced penile squamous cell carcinoma (PSCC).

**Objectives:**

To determine the prevalence of PIK3CA copy number gain and correlate this with the activity status of PI3K-AKT-mTOR pathway in pre-malignant penile intraepithelial neoplasia (PeIN) and invasive PSCC.

**Materials and methods:**

Archival tissue blocks were obtained from 58 PeIN and 244 primary PSCC patients treated at St George’s Hospital. PIK3CA copy number status (CNS) was assessed by fluorescence *in-situ* hybridisation. High-risk HPV DNA was detected with INNO-LiPA assay. p16^INK4A^, p-AKT and p-mTOR protein expression were assessed using immunohistochemistry (IHC).

**Results:**

Increased prevalence of PIK3CA copy number gain was seen in PSCC in comparison to PeIN (84/199 (42%) vs. 10/58 (17%); p = 0.0009). Analysis of the p-AKT and p-mTOR revealed a tendency to a more common expression of cytoplasmic p-AKT (p = 0.1318), nuclear p-AKT (p<0.0001) and cytoplasmic mTOR (p = 0.0006) in PeIN than PSCC. A significant association between p-AKT cytoplasmic immunoexpression and PIK3CA CNS (p = 0.0404) was found in PeIN.

**Conclusion:**

Overall, PIK3CA copy number gain correlated with activation of the PI3K-AKT-mTOR pathway in PeIN and activation of this pathway is primarily involved in early penile carcinogenesis. Based on these results therapeutic targeting of this pathway in advanced PSCC is unlikely to produce significant clinical benefit. Future studies will need to focus on alternative therapeutic targets.

## Introduction

Penile squamous cell carcinoma (PSCC), though uncommon with an incidence of less 1 per 100,000 men in Europe and USA [1], poses significant physical and psychological stress to those afflicted [2]. Risk factors for PSCC include high-risk human papilloma virus (hr-HPV), phimosis, lichen sclerosis and smoking among others [1, 2]. A multitude of different histological subtypes of PSCC exist with distinct clinical and prognostic associations [3].

With PSCC two distinct patient groups exist, the first a large cohort with a high cure rate (80%) and excellent long-term survival in those with organ-confined disease with no or minimal regional lymph node involvement [2]. The second is a smaller patient cohort with highly aggressive disease with significant propensity to metastasise and poor prognosis [4]. Management of the latter group with aggressive and advanced tumours still remains difficult as most are chemo/radio-resistant with limited treatment options available when first line options fails [5].

The key molecular alterations driving PSCC development and potential therapeutics targets are incompletely understood. The phosphatidylinositol-4,5-bisphosphate 3-kinase, catalytic subunit alpha (PIK3CA) gene is mutated and/or amplified/gained in numerous cancers, leading to dysregulation of the PI3K-AKT-mTOR pathway and resulting in increased cell proliferation, angiogenesis and survival [6]. Previous mutational analysis papers [7] and more recent next-generation sequencing studies in PSCC have confirmed the involvement of the altered PIK3CA oncogene in penile carcinogenesis [8, 9].

Few papers have looked into the activity status of this pathway in PSCC, one of which was Ferrandiz-Pulido *et al*. [10] whom in 67 PSCC cases found p-mTOR (activated mTOR) and p-elF4E (a downstream effector protein of mTOR) immunoexpression was significantly increased in PSCC compared to adjacent normal tissues and associated with lymph node metastasis (p = 0.05 and p = 0.006, respectively). Raising the possibility of using already established biological therapies such as everolimus and temsirolimus (mTOR inhibitors), to target this pathway in PSCC [11].

Our objective was to determine the prevalence of PIK3CA copy number gain and correlate this with the activity status of PI3K-AKT-mTOR pathway in different disease states of penile cancer via utilisation of a large cohort of both pre-cancerous penile intraepithelial neoplasia (PeIN) and invasive PSCC cases.

## Materials and methods

### Ethical approval

Ethics approval for this study was obtained and approved by East London and The City Research Ethics Committee (The Orchid Tissue Bank: 09/H0704/04). All patients provided written, informed consent to have their medical record and their tissue for use in this research.

### Patients

We retrospectively retrieved 58 PeIN and 244 primary PSCC archival formalin-fixed paraffin-embedded (FFPE) blocks from patients treated at St George’s Hospital, London. All cases were reviewed by a panel of expert uropathologists (CC and BT) including tumour subtyping, grading, staging and lymph node status via standard methodologies.

### Fluorescence *in-situ* hybridisation

PIK3CA copy number status (CNS) was assessed using two BAC clones for FISH probe preparation, which were obtained from the Institute of Cancer Research (Sutton, UK). The first clone (RP11-348P10) was for the control 3p21.3 region and was and the second clone (RP11-245C23) for the gene of interest, PIK3CA located at 3q26.3. The distal 3p21.3 locus was chosen as the control, as this locus showed no copy number change on our previous array comparative genomic hybridisation data utilising the same PSCC cohort [12]. The PIK3CA gene probe was labelled red and the control gene probe was labelled green.

Tissue microarrays (TMA) were prepared with a manual microarrayer using obtained archival FFPE tissue blocks. Three x 1 mm tissue cores were taken from each tumour. TMA slides were de-waxed in xylene, washed in ethanol and water. Next, they were boiled in pre-treatment buffer (Spotlight tissue pre-treatment kit, Invitrogen) for 15 minutes and digested with pepsin solution (Digest All-3, Invitrogen) for 5 minutes at 28°C. After washing in water and air-drying the slides, the probe was applied following the manufacturer instructions and slides were denatured at 98°C for 10 minutes. The slides were then hybridised at 37°C for 24 hours. A post-hybridization wash in 2 x SCC buffer for 5 min at 42°C was performed, followed by PBS washes. Slides were then dried, counterstained and mounted with Vectashield antifade solution containing DAPI (Vector Laboratories LTD).

All TMA FISH slides were scanned and analysed using the Ariol SL-50 System (Applied Imaging, San Jose, CA, USA). A minimum of 100 cells with clear hybridization signals was counted per sample. On the basis of FISH results in normal penile epithelium, primary PSCC samples were considered to have copy number gain if the number of FISH signals for PIK3CA was greater than for the normal control probe in at least 20% of counted nuclei. No signal clusters specific for gene amplification were present. Cores with high background or very weak signals were excluded from the analysis.

### HPV analysis

The presence of HPV DNA was detected by PCR method using SPF10 primers, which amplify a 65-bp fragment of the conserved L1 open reading frame and HPV genotypes identified by the INNO-LiPA HPV Genotyping Extra Assay (Innogenetics NV, Ghent, Belgium) as previously described [13] in 216 PSCC cases. Due to the presence of poor quality DNA from 28 FFPE primary PSCC samples, HPV detection and genotyping was not possible.

### Immunohistochemistry

Four-millimeter sections were cut and immunostained using standard heat-induced antigen retrieval methods (pressure cooking for 11 min) with citrate low pH buffer and the ABC kit (Vector Laboratories, PK-6200). Primary monoclonal antibodies were applied as follow: p16^INK4A^, 1:800 (Santa Cruz, 56330, mouse); p-AKT, 1:60 (Cell Signalling, 3787, rabbit, Ser473); p-mTOR, 1:60 (Cell Signalling, 2976, rabbit, Ser2448). Positive controls included penile cancer for p16^INK4A^, prostate cancer for p-AKT and normal pancreas for p-mTOR. For negative control slides, primary antibody step was omitted and only antibody diluent applied instead.

IHC sections were scored semi- quantitatively using a common scoring scheme by two consultant genitourinary pathologist (GT and DB), separately, without any knowledge of the clinical data. Any scoring discrepancies were resolved by both pathologists re-analysing these section(s)/core(s) under a dual-headed light microscope and coming to a joint consensus. All proteins, p16^INK4A^, p-AKT and p-mTOR showed both cytoplasmic and nuclear staining patterns. The intensity of cytoplasmic and nuclear staining was measured as: 0 (nil), 1 (weak), 2 (moderate) and 3 (strong). The final cytoplasmic score was from 0–3. For nuclear positivity each core was given an estimated visual score between 0–100% representing the percentage of positively stained nuclei. The final nuclear score was deduced by multiplying the percentage of stained nuclei by intensity (0–3) to give an expression score from 0 to 300.

Cores which were lost or damaged during the experimental process were excluded from the analysis. The core with the highest score was selected for analysis. Each core was given a score for cytoplasmic and nuclear staining for p16^INK4A^, p-AKT and p-mTOR. For nuclear staining any nuclear expression score >0 was considered to be positive for both p-AKT and p-mTOR [13]. For cytoplasmic staining we classified an intensity score of 0 or 1 as negative and a score of 2 or 3 as positive for p-AKT and p-mTOR [14]. A cytoplasmic and nuclear intensity score of 3 (median value) was considered as positive for p16^INK4A^ immunostaining and thus hr-HPV positive. The association between p-AKT and p-mTOR and our histopathological data was also evaluated.

### Statistical analysis

Statistical analysis was performed using GraphPad Prism Version 5.03 for Windows. Statistical tests included Chi-squared test (X^2^ test) or Fisher’s exact test (FET) for PIK3CA CNS comparison with hr-HPV status, IHC results and histopathological data. Spearman’s rank correlation test (SRC) was used to assess the correlation between the IHC expressions of the investigated proteins, p-AKT and p-mTOR. All analyses were two sided, p<0.05 were considered significant.

## Results

### PIK3CA copy number status

**Fig 1:**
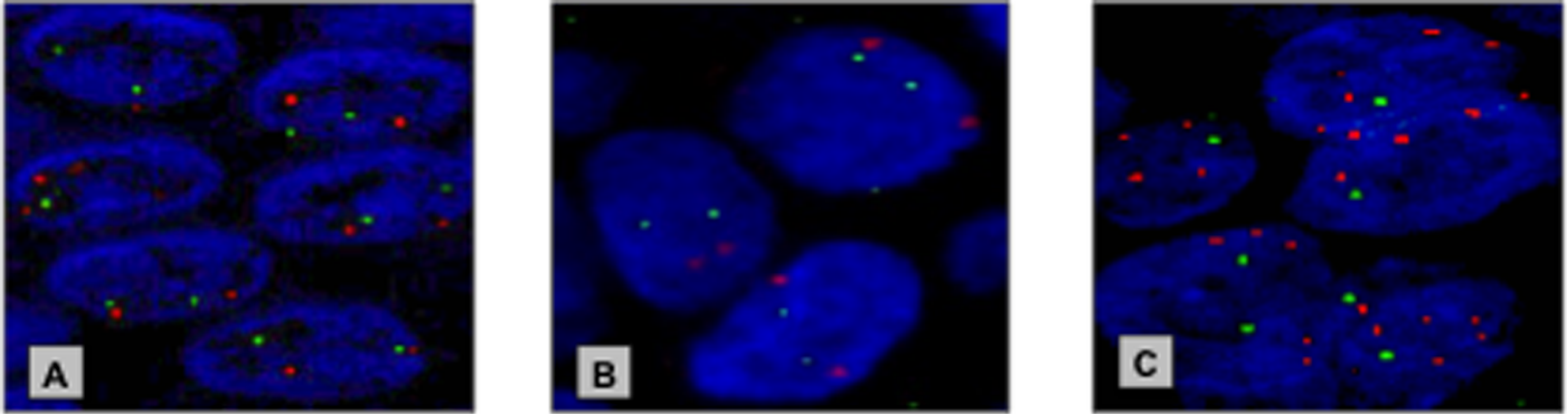
Example of PIK3CA (3q26.3) copy number gain detected by FISH. **(A)** normal copy number at the *PIK3CA* locus in normal penile epithelium (**B)** normal copy number of the *PIK3CA* locus in PeIN (**C)** copy gain of the PIK3CA locus in primary PSCC. *PIK3CA* locus—designated by the red probe & Control—designated by the green probe.

We analysed 58 patients with PeIN and 244 patients with primary PSCC with a median age of 65 (range 32–84) and 66 (range 32–91), respectively. PIK3CA CNS assessment was possible in 58 PeIN and 199 primary PSCC cases. PIK3CA copy number gain was detected in 10/58 (17%) and 84/199 (42%) of PeIN and primary PSCC cases, respectively. Increased prevalence of PIK3CA copy number gain in cases with invasive PSCC was statistically significant (p = 0.0009, X^2^ test). (Fig 1)

### HPV analysis

**Fig 2:**
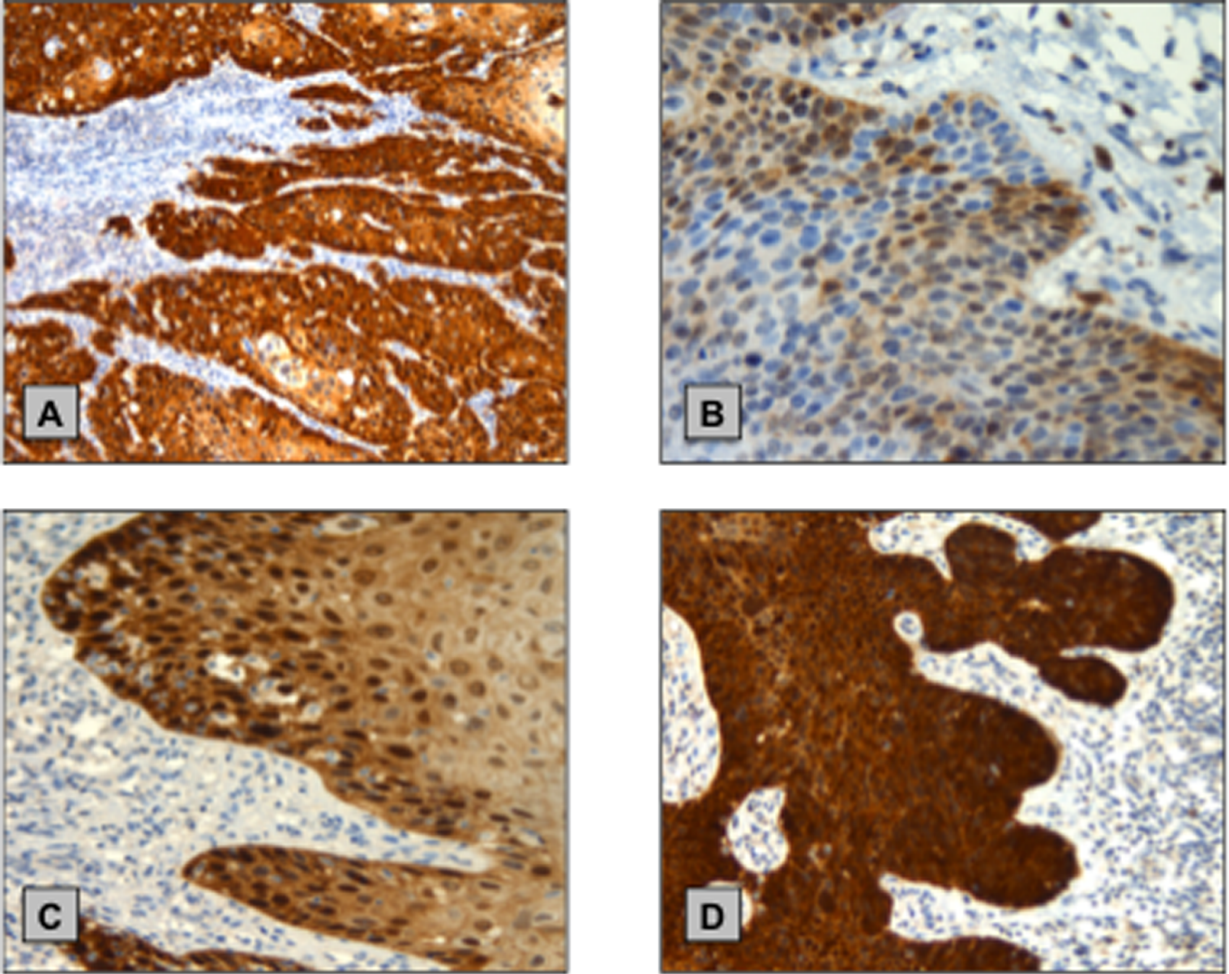
Example of p16^INK4A^ IHC staining in PeIN and primary PSCC Samples. **(A)** Positive control—primary PSCC with HPV infection detected by INNO-LiPA assay **(B)** PeIN with weak cytoplasmic and nuclear staining (intensity score 1—classified as negative) **(C)** PeIN with moderate cytoplasmic and nuclear staining predominantly localized in the basal layer (intensity score 2—classified as negative) **(D)** PeIN with strong, uniform and confluent cytoplasmic and nuclear staining (intensity score 3—classified as positive).

High risk HPV DNA was detected in 45/58 (78%) and 121/216 (56%) of PeIN and primary PSCC cases, respectively (Fig 2). High-risk HPV 16 was the most prevalent type, seen in 100/121 (83%) of hr-HPV positive PSCC. The difference between the hr-HPV rates between the PeIN and PSCC group was statistically significant (p = 0.0038, X^2^ test). (Table 1)

**Table 1:** PIK3CA CNS and Hr-HPV status in PeIN and primary PSCC.

	PeIN (%)	PSCC (%)	X^2^ test (p Value)
**PIK3CA CNS**			
**No Gain**	48 (83)	115 (58)	0.0009
**Gain**	10 (17)	84 (42)	
**Hr-HPV Status**			
**Negative**	13 (22)	95 (54)	0.0038
**Positive**	45 (78)	121 (46)	

HPV status and PIK3CA CNS were both available for 58 cases of PeIN and 178 cases of primary PSCC. No significant difference in the frequency of PIK3CA copy number gain between hr-HPV positive and negative PSCC was found in the PeIN and primary PSCC cohorts (p = 1 and p = 0.1172, respectively, X^2^ test). (Table 1)

### Phosphorylated AKT and mTOR immunohistochemistry

**Fig 3:**
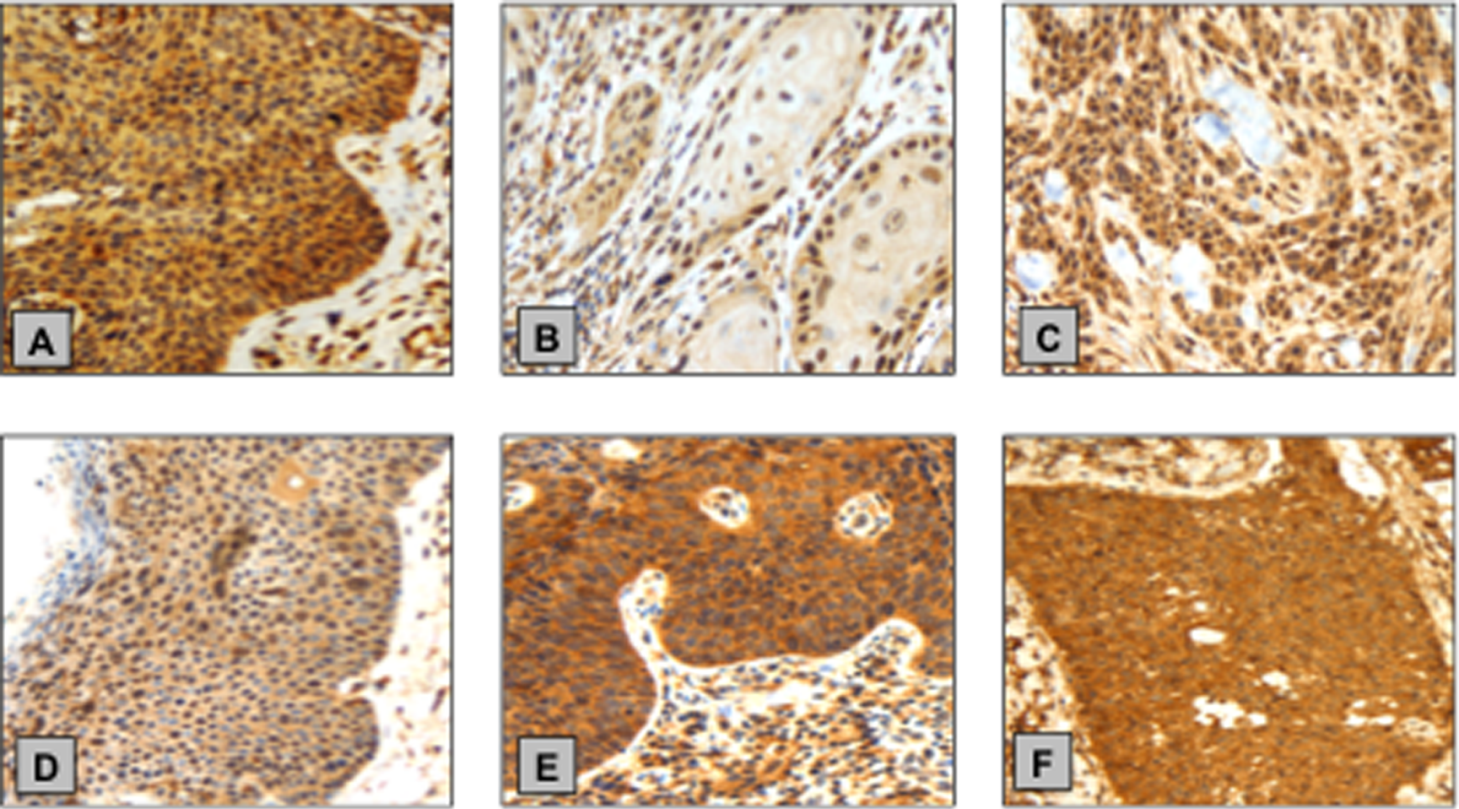
Example immunoexpressions of p-AKT and p-mTOR in PeIN and PSCC. **(A)** PeIN specimen with strong p-AKT cytoplasmic staining and moderate nuclear staining **(B)** PSCC with weak p-AKT cytoplasmic staining **(C)** Primary PSCC with moderate p-AKT cytoplasmic and nuclear staining (**D)** PeIN tissue with weak p-mTOR cytoplasmic staining and moderate nuclear staining **(E)** PeIN specimen with strong p-mTOR cytoplasmic staining **(F)** Primary PSCC with strong p-mTOR cytoplasmic staining.

Examples of p-AKT and p-mTOR immunostaining in PeIN and PSCC cases are shown in Fig 3. PeIN cases (23/58; 40%) had more positive p-AKT cytoplasmic expression than primary PSCC cases (70/238; 29%) however this was not statistically significant (p = 0.1318, X^2^ test). p-AKT nuclear expression was more common in PeIN cases (39/58; 67%) than primary PSCC cases (76/238; 32%) and this was statistically significant (p = 0.0001, X^2^ test).

Similarly, we found PeIN cases (29/58; 50%) had significantly more positive p-mTOR cytoplasmic expression than primary PSCC cases (64/239; 27%) (p = 0.0006, X^2^ test). Conversely, PeIN had a lower percentage of cases with positive nuclear p-mTOR immunoexpression in comparison to primary PSCC cases (11/58; 19% vs. 64/239; 27%), however this was not significant (p = 0.2192, X^2^ test).

In both the PeIN and primary PSCC cohorts’ cytoplasmic and nuclear p-AKT were found to significantly correlate with cytoplasmic and nuclear p-mTOR (p<0.0001 and p = 0.0043 vs. p<0.0001 and p = 0.0402, respectively, SRC). A significant positive association between PeIN p-AKT cytoplasmic immunoexpression and PIK3CA copy number gain was found (p = 0.0404, FET), however this was not the case for our primary PSCC cohort (p = 0.3229, X^2^ test). No other significant associations between PIK3CA CNS and nuclear p-AKT, cytoplasmic and nuclear p-mTOR immunoexpression were identified.

p-AKT and p-mTOR protein immunoexpression in relation to PSCC histopathological characteristics are shown in Tables 2 and 3. A significant association was seen between both positive expressions of cytoplasmic p-AKT and p-mTOR with a more aggressive tumour subtype (p<0.0001 and p = 0.0005, respectively, X^2^ test) and higher grade (p = 0.007 and p = 0.0103, respectively, X^2^ test). Contrastingly, nuclear p-AKT expression was just significantly associated with a negative lymph node status (p = 0.0409, X^2^ test). Similarly, nuclear p-mTOR was significantly associated with a lower tumour grade (p = 0.0053, X^2^ test).

**Table 2:** Correlation of p-AKT immunoexpression with PSCC histopathological characteristics.

	p-AKT^C^ Score (%)		p Value	p-AKT^N^ Score (%)		p Value
	Negative	Positive		Negative	Positive	
**Tumour Subtype**						
**Verrucous**	31 (15)	6 (3)		31 (15)	6 (3)	
**Usual**	104 (50)	45 (21)	<0.0001^**§**^	99 (47)	50 (24)	0.0654^**§**^
**Basaloid**	7 (3)	17 (8)		14 (7)	10 (4)	
**Tumour Grade**						
**1**	38 (16)	5 (2)		30 (13)	13 (5)	
**2**	57 (24)	22 (9)	0.007^**§**^	53 (22)	26 (11)	0.955^**§**^
**3**	73 (31)	43 (18)		79 (33)	37 (16)	
**Tumour Stage**						
**I**	51 (22)	25 (10)		45 (19)	31 (13)	
**II**	78 (33)	33 (14)	0.6037^**§**^	76 (32)	35 (15)	0.0575^**§**^
**III+IV**	37 (16)	12 (5)		39 (17)	10 (4)	
**Nodal Status**						
**Negative**	96 (45)	42 (20)	0.5631^**§**^	90 (42)	48 (23)	0.0409^**§**^
**Positive**	55 (26)	20 (9)		59 (28)	16 (7)	

^C^—cytoplasmic expression;

^N^—nuclear expression;

^§^ - Statistical analysis performed using X^2^ test

**Table 3:** Correlation of p-mTOR immunoexpression with PSCC histopathological characteristics.

	p-mTOR^C^ Score (%)		p Value	p-mTOR^N^ Score (%)		p Value
	Negative	Positive		Negative	Positive	
**Tumour Subtype**						
**Verrucous**	33 (16)	3 (1)		23 (11)	14 (7)	
**Usual**	106 (51)	44 (21)	0.0005^**§**^	110 (52)	40 (19)	0.092 ^ϑ^
**Basaloid**	11 (5)	13 (6)		21 (10)	3 (1)	
**Tumour Grade**						
**1**	39 (16)	5 (2)		28 (12)	16 (7)	
**2**	60 (25)	19 (8)	0.0103^**§**^	51 (21)	28 (12)	0.0053^**§**^
**3**	76 (32)	40 (17)		96 (40)	20 (8)	
**Tumour Stage**						
**I**	54 (23)	23 (10)		52 (22)	25 (11)	
**II**	81 (34)	30 (13)	0.6581^**§**^	83 (35)	28 (12)	0.3946^**§**^
**III+IV**	38 (16)	11 (4)		38 (16)	11 (4)	
**Nodal Status**						
**Negative**	106 (55)	33 (17)	0.4937^**§**^	102 (53)	37 (19)	0.6758^**§**^
**Positive**	21 (11)	33 (17)		18 (9)	37 (19)	

^**C**^**—**cytoplasmic expression;

^**N**^**—**nuclear expression;

^**§**^ **-** Statistical analysis performed using X^2^ test;

^ϑ^**-** Statistical analysis performed using FET

## Discussion

To our knowledge, this is the first study to evaluate the prevalence of PIK3CA copy number aberration and activation of the PI3K-AKT-mTOR pathway in early stage, premalignant PeIN and invasive primary PSCC cohorts. A significant trend was seen with an increasing frequency of PIK3CA copy number gain in those with invasive PSCC disease (p<0.0001): PeIN 10/58 (17%) and primary PSCC 84/199 (42%).

The presence of PIK3CA copy number gain in a limited number of PeIN suggests it to be an early event in penile carcinogenesis, which becomes enriched with cancer progression. It is likely that PIK3CA copy number gain is strongly selected for at multiple stages of penile carcinogenesis, and plays a role both in early stage disease and the establishment of a malignant phenotype. Heselmeyer *et al*. demonstrated a similar pattern in CSCC as they found that primary CSCC (9/10; 90%) and advanced stage (≥ Stage T2b) primary CSCC (23/30; 77%) exhibited greater copy number gain in chromosome 3q than severe dysplastic (1/13; 8%) specimens [15, 16].

Analysis of the PI3K-AKT-mTOR pathway in different disease states of penile cancer revealed more common expression of nuclear p-AKT and cytoplasmic p-mTOR in PeIN than primary PSCC (p<0.0001 and p = 0.0006, respectively). Both cytoplasmic and nuclear AKT are known to be involved in anti-apoptotic and pro-proliferative cellular activities [17] and cytoplasmic mTOR is known to play a key role in protein translation initiation [18]. In addition, a significant association between PeIN p-AKT cytoplasmic immunoexpression and PIK3CA copy number status (p = 0.0404) was found.

Overall, it appears that early stage PeIN disease has greater activation of the PI3K-AKT-mTOR pathway than invasive primary PSCC. These findings agree with Pedrero *et al*. [19] who found that PIK3CA amplification is an early event in HNSCC in 43/117 (37%) cases, while Chaux *et al*. [13] analysed 112 PSCC cases and found low levels of p-AKT (Ser473) and p-mTOR (Ser2448) immunoexpression. Our results propose that this pathway likely plays a role in early penile carcinogenesis and on the advancement of PSCC alternative pathways play a more significant role, with the PIK3CA oncogene and PI3K-AKT-mTOR pathway having a lesser role and as such targeted therapeutics with current mTOR inhibitors in advanced PSCC are unlikely to produce widespread significant clinical benefit, without careful patient selection.

Despite the association of PIK3CA copy number gain and activation of the PI3K-AKT-mTOR pathway with early stage penile carcinogenesis, an increased frequency of PIK3CA copy number gain with invasive PSCC was identified. It is possible that the increased frequency of PIK3CA copy number gain seen in primary and advanced primary PSCC is a by-product of increasing genomic instability with cancer progression [20]. However, the presence of 3q gain, in multiple HPV induced cancers [21] and its association with reduced cancer-specific and disease-free survival in PSCC [22], implies this region has an important role in late stage disease progression such as invasion and metastasis, suggesting that it possibly harbours other yet unidentified drivers of penile cancer progression.

Various distinct histological subtypes of PSCC exist with differing metastatic potential and prognosis, this study looked primarily at the slow growing and low-grade verrucous subtype, the more common usual subtype and the more clinically aggressive with a high metastatic potential, basaloid histotype [3]. p-AKT and p-mTOR cytoplasmic expression in primary PSCC correlated with both a higher tumour grade and a more aggressive tumour subtype, while their nuclear expression associated with negative lymph node status and a lower tumour grade, respectively. Murayama *et al*. [23] and Stankiewicz *et al*. [24] found similar results with cytoplasmic p-mTOR immunoexpression in gastric cancer and with nuclear p-AKT immunoexpression in PSCC, respectively. In addition, Ferrandiz-Pulido *et al*. [10] found cytoplasmic p-mTOR immunoexpression to be associated with aggressive disease. These findings suggest that the localisation of activated AKT and mTOR may effect tumour progression.

Rosner *et al*. found that the activity of AKT is significantly higher in the cytoplasm than in the nucleus and that mTOR, a central regular of cellular metabolism and biosynthesis, is also predominately located in cytoplasm [25, 26]. The increased expression and activity of these phospho-proteins in the cytoplasm in comparison to nucleus may explain our results.

Hr-HPV infection was found in 45/58 (78%) of PeIN, and in 121/216 (56%) of PSCC. High HPV prevalence in premalignant PeIN was also reported by Mannweiler *et al*. [27] who noted 33/43 (77%) premalignant PeIN cases demonstrated both HPV infection and p16^INK4A^ overexpression in their cohort. Prevalence of hr-HPV infection in our PSCC cohort correlates well with previous reports [24, 28]. A significant variation in the proportion of PSCC carrying hr-HPV DNA is evident in the literature and this is likely due to the disparity in HPV detection methods employed, population differences and PSCC histological subtypes included [28].

The main limitation of this study was the relatively small number of cases available for analysis and this is a well-documented issue and constraint when performing research on rare malignancies such as PSCC. However, our observations, though negative, are important due to the lack of research in this field and need for additional therapeutic options.

## Conclusion

PIK3CA copy number gain is associated with activation of the PI3K-AKT-mTOR pathway in PeIN and activation of this pathway is primarily involved in early penile carcinogenesis. The increased frequency of PIK3CA copy number gain seen in invasive primary PSCC is not reflected in elevated PI3K-AKT-mTOR pathway activation. Therefore, it is likely a result of increased genomic instability with cancer progression. Based on our results the therapeutic targeting of the PI3K-AKT-mTOR pathway in those with advanced PSCC is unlikely to produce significant clinical benefit. Larger future studies including functional analysis are needed to corroborate these findings in addition to discovering other promising therapeutic targets.
